# The efficacy of a Mindfulness Based Intervention for depressive symptoms in patients with Multiple Sclerosis and their caregivers: study protocol for a randomized controlled clinical trial

**DOI:** 10.1186/s12883-016-0528-0

**Published:** 2016-01-13

**Authors:** Sara Carletto, Martina Borghi, Diana Francone, Francesco Scavelli, Gabriella Bertino, Marco Cavallo, Simona Malucchi, Antonio Bertolotto, Francesco Oliva, Luca Ostacoli

**Affiliations:** Clinical Psychology and Psychosomatics Service, A.O.U. San Luigi Gonzaga, University of Turin, Regione Gonzole 10, Orbassano, TO 10043 Italy; Neurologia 2—CRESM (Centro Riferimento Regionale Sclerosi Multipla), AOU San Luigi, Regione Gonzole 10, Orbassano, TO 10043 Italy; eCampus University, Via Isimbardi, Novedrate, Como 10-22060 Italy; Department of Mental Health, Azienda Sanitaria Locale Torino 3, Via Martiri XXX Aprile, Collegno, Torino 30-10093 Italy; Clinical and Biological Sciences Department, University of Turin, A.O.U. San Luigi Gonzaga, Regione Gonzole 10, Orbassano, TO 10043 Italy

**Keywords:** Multiple Sclerosis, Depression, Quality of Life, Mindfulness, Mindfulness Based Intervention, Psycho-education, Relaxation, Caregiver

## Abstract

**Background:**

Multiple Sclerosis has a great impact on psychological functioning of patients and can be associated with various mental health disorders and symptoms. The most prevalent one is depression, which ranges from 15 to 47 %.

Mindfulness Based Interventions are a relatively brief and cost-effective program that has been studied in patients with several chronic diseases and recently also in patients with Multiple Sclerosis. Mindfulness Based Interventions are based on the assumption that a non-judgmental awareness and acceptance of one’s moment-to-moment experience can have a positive effect on the adaptation to the disease, reducing the psychological burden and improving patients’ quality of life.

Several studies concluded that Mindfulness Based Interventions can be beneficial in terms of improving both psychological and psychical aspects of Multiple Sclerosis, but none of them compared the intervention with an active control group. The primary objective of the study is to evaluate the efficacy of a group-based Mindfulness Based Intervention on depressive symptoms in patients with Multiple Sclerosis, as compared with an active control group.

**Methods:**

The study design is a randomized controlled clinical trial. Eighty-eight patients with Multiple Sclerosis and depressive symptoms will be recruited and randomized to either Mindfulness Based Intervention or an active control group. The latter is designed to control for non-specific elements of the intervention and it comprises psycho-education and relaxation techniques. The primary outcome is the reduction of depressive symptoms as measured via the Beck Depressive Inventory-II. Secondary outcome measures are level of quality of life, anxiety, perceived stress, illness perception, fatigue and quality of interpersonal relationship. Outcomes will be assessed at baseline, after treatment and 6 months after the end of the treatment. Caregivers will participate in groups together with patients.

**Discussion:**

As far as we know this trial will be the first randomized controlled trial testing the efficacy of group-based Mindfulness Based Intervention for patients with Multiple Sclerosis with a comparison with an active control group with a specific focus on depressive symptoms.

**Trial registration:**

NCT02611401.

## Background

Multiple Sclerosis (MS) is a chronic demyelinating disease with an onset typically during young age, that poses a significant emotional burden with heavy psychosocial consequences. People with MS have to deal with the unpredictability of the disease, the loss of function and disability, a series of debilitating and changeable symptoms and the uncertain perspectives of the disease. The physical limitations of the disease have also a great impact on social functioning.

Previous studies have focused on the psychosocial impact of MS, showing a high prevalence of depression, anxiety and a reduced Quality of Life (QoL) [1–6]. Depression affects from 15 to 47 % of patients [7, 8], with lifetime prevalence estimates at 50 % [9]. This prevalence is much higher than in other chronic neurological diseases [1] and is three times higher than in the general population [4].

There is a strong correlation between fatigue and depression [10] and also between depression and anxiety [11], and these interactions have important implications for QoL [1].

The Goldman Consensus Conference Study Group on depression in MS stated that affective disorders are not usually recognized by clinicians, and do not routinely receive treatment [12]. More recently, the American Academy of Neurology [13] formulated evidence-based recommendations for screening, diagnosing and treating psychiatric disorders in individuals with MS and recommended future research on the effectiveness of treatments frequently used in the non-MS population.

Furthermore, a recent study on psychological treatment of depressive symptoms in patients with medical disorders stated that treating comorbid depression should be one of the priorities in medical care settings [14]. Feinstein [4, 15] highlighted that treatments for depression in public health care services may also be adjusted according to the availability of resources and access to them. Thereby it is essential to establish brief and cost-effective interventions to reduce depression symptoms and the psychological burden and to improve QoL of MS patients [15].

To our knowledge, so far there are only few studies with an adequate methodology that investigated the efficacy of psychopharmacologic treatment for depression in MS. A Cochrane Review [16] showed that Serotonin Selective Reuptake Inhibitors are quite effective, but with prominent side effects and high drop-out rates. Several studies of psychological interventions have shown benefits upon depression and QoL of life in patients with MS. A Cochrane review [17] and a recent meta-analysis [18] identified that Cognitive Behavioural Therapy (CBT) had a moderate effect on depression.

Recently, group intervention programs have been proposed to improve the well-being and health status of patients with chronic illnesses, with the aim of reducing distress and social isolation related to the disease by means of a group sharing support. In particular, Mindfulness Based Interventions (MBIs) are a relatively brief and cost-effective program that has been employed effectively in patients with several diseases, such as chronic pain, cancer, fibromyalgia [19] and also recently in patients with MS [20, 21].

MBIs are based on the premise that an attitude of non-judgmental awareness and acceptance of the present moment have an effect on the distressing tendencies to escape from or to over-engage with one’s disturbing feelings, emotions and thoughts. Mindfulness can positively have an effect on coping strategies and on the adaptation to the disease, by encouraging patients to relate differently to their physical and psychological symptoms [4].

Several neuroimaging studies and of biological markers [22–24] had also pointed out the effects of mindfulness at a neurobiological level, showing its effects on the regulation of emotional responses through the inhibition of amygdala by a proper functioning of the prefrontal cortex. MBIs efficacy was also associated with an altered cortisol and immune patterns consistent with less stress and mood disturbance, and decreased blood pressure.

In recent years, several studies provided support for the positive association of mindfulness with both emotion regulation and QoL in MS patients. Senders et al. [25] found that trait mindfulness is significantly associated with decreased psychological stress, a more constructive coping profile, increased resilience and better QoL in MS. The study of Schirda et al. [26] corroborated these results, highlighting that reduced emotion dysregulation may be a critical pathway linking mindfulness and QoL in MS, especially in those with higher symptoms of depression.

Recently two systematic reviews [20, 21] focused on the efficacy of MBIs in MS patients, concluding that MBIs can be beneficial, especially in terms of improving the QoL, mental health parameters (such as anxiety and depression) and physical parameters (as fatigue). Similar results were recently obtained also in a pilot randomized controlled trial in patients with Progressive MS [27]. Furthermore, MBIs were shown to have a broad feasibility and satisfaction, a low attrition rate and no side effects [20, 28].

The major limit pointed out by all these studies is the absence of a comparison of MBI intervention with an active control group. Both reviews claimed for additional rigorous clinical trials with larger sample size and with robust methodological techniques [20, 21].

In order to overcome this limit, in our randomized controlled trial we will include an active control intervention. Moreover, we aim to evaluate the efficacy of the MBI in MS patients who have depressive symptoms. In fact, NICE guidelines [29] indicated that psychological interventions such as mindfulness or psycho-education, combined with drug therapies or alternative to them depending on the case, resulted more effective both in terms of efficacy and in term of cost-effectiveness compared to drug therapy alone. As highlighted by previous study [19, 26], MBIs may be more effective and useful in populations with mood disorders. Furthermore, our project aims to involve patients’ caregivers asking them to participate in the group the patient has been assigned to. We expect that caregivers will benefit from the treatment received, due to the load of stress and difficulties related to the management of a chronic disabling diseases like MS.

The aim of the current paper is to describe our research design. Our primary objective is to evaluate the efficacy of MBI on depressive symptoms of MS patients, compared to an active control intervention, and to assess whether these expected benefits can be maintained at the 6-month follow-up. Secondary aims of the study are: to assess whether after the Mindfulness Based Intervention there is an improvement in QoL, anxiety, fatigue and perceived stress levels; to evaluate the efficacy of the intervention on the perception of the illness and whether participating in MBI also improve interpersonal relationships; to evaluate the efficacy of MBI on depression, QoL and related symptoms in caregivers.

We hypothesize that MBI intervention will lead to a reduction of depressive symptoms, and its associated symptoms both at a physical and psychological levels, compared to an active control group.

## Methods

### Design

This study is a randomized controlled clinical trial. The study is registered in ClinicalTrials.gov registry as NCT02611401.

### Setting

Participants will be enrolled at the CReSM Unit (Regional Reference Centre for Multiple Sclerosis), affiliated with the San Luigi University Hospital of Orbassano, Italy, which includes about 1500 patients with MS.

The study protocol is approved by the Medical Ethics Committee of San Luigi Gonzaga University Hospital.

### Participants

The subjects of the study will be 88 patients with MS and depressive symptoms that will be pre-screened from a catchment group of about 600 patients using the Beck Depression Inventory-II (BDI-II). Those who have a score on BDI-II greater than 13 (considered the best approach to screening for depression in MS patients following the Goldman Consensus Group) [12] will be assessed with the other psychological measures.

Inclusion criteria are as follows:—definite diagnosis of MS (Mc Donald Criteria) [30] evaluated by a neurologist of the CReSM Unit at least 6 months prior the beginning of the study;—relapsing-remitting and secondary progressive disease;—age of 18–65 years;—clinically inactive phase of the disease;—an Expanded Disability Status Scale (EDSS) score lower than 6.5;—fluent Italian speaker;—legal capacity to consent to the treatment;—willingness to abstain from or to suspend all concomitant psychological treatment;—suspension of all psychotropic medications at least 1 month before the treatment or maintenance at baseline level throughout the study.

Exclusion criteria are as follows:—current severe Major Depressive Disorder (as assessed by Structured Clinical Interview-DSM-IV (SCID);—severe suicidality, including ideation, plan, and intent;—current serious psychological and psychiatric disorders, including psychotic disorders, bipolar disorders, active substance abuse (as assessed by the SCID);—presence of overt dementia;—corticosteroid treatment during the previous 30 days;—other serious medical disorders in addition to MS;—current pregnancy.

The research protocol will be proposed to patients who will meet the inclusion criteria, with an explanation of the aims of the study and declaring the possibility that they are assigned by random allocation to MBI group or to control group. Patients who will give informed consent will be included in the study.

### Randomization and blinding

Patients will be randomly allocated to one of the two conditions: MBI and active control group. Patients will be randomized to the intervention or control group with a 1:1 ratio, using a block-wise randomization sequence (block size of 4 and 6).

The sequence will be determined by an independent researcher, blind to initial assessment to ensure allocation concealment, using a random number generator (www.randomizer.org).

Treatment assignment will be communicated to the patients by the study coordinator to ensure the blinding of the evaluators.

One family member or caregiver for each patient is asked to join the group the patient has been assigned to. Because it is not possible to stratify for this variable, and as it is not our primary outcome, we expect that due to randomization the proportion of caregivers participating in the study will be distributed randomly and equally in both groups.

### Assessments

All patients in the care of the CReSM are evaluated by neurologists with the EDSS (Expanded Disability Status Scale). The psychological assessment will be performed with the same timing and tools: at baseline (T0), after treatment (T1) and 6 months after the end of the group intervention (T2). The psychological assessment of the patients entering the study will be performed by trained Clinical Psychologists with more than 3 years of clinical practice in the liaison setting. The psychological assessment will be independent and blind to treatment, and will encompass the administration of the following clinical interview and self-report questionnaires.

- Structured Clinical Interview-DSM-IV (SCID-IV) [31]: is a well-validated, semi-structured interview, which covers the Diagnostic and Statistical Manual of Mental Disorders, 4th Edition (DSM-IV) criteria for adult psychopathology.
- Beck Depression Inventory-II (BDI) [32]: it is a 21-item self-report instrument that assesses the presence and severity of symptoms consistent with the criteria of the DSM-IV. Each item is scored on a 4 point scale ranging from 0 to 3 with total scores ranging from 0 to 63, where higher scores are indicative of higher levels of depression. The Consensus Group for depression in MS [12] and the American Academy of Neurology [13] stated that the best approach to screening for depression in general MS populations is to use the BDI-II, with a cut-off score of 13.
- Functional Assessment of Multiple Sclerosis (FAMS) [33]: it is a self-report scale designed to assess six primary aspects of QOL of patients with MS: Mobility, Symptoms, Emotional Well-Being, General Contentment Thinking and Fatigue, and Family/Social Well-Being.
- Beck Anxiety Inventory (BAI) [34]: is a 21-item self-report measure that assesses cognitive, somatic, and affective anxiety symptom severity. Each item is rated on a Likert scale from 0 to 3, and the total BAI score can range from 0 to 63, where higher scores are indicative of higher levels of anxiety.
- Fatigue Severity Scale (FSS) [35]: is a self-administered questionnaire with 9 items investigating the severity of fatigue in different situations during the past week. This items are designed to differentiate fatigue from clinical depression.
- Inventory of Interpersonal Problems (IIP) [36]: is a self-report instrument that identifies a person’s most salient interpersonal difficulties. The IIP contains 64 statements describing common interpersonal problems.
- Brief Illness Perception Questionnaire (IPQ-Brief) [37]. It is a nine item self-report measure that is used to assess the major components of illness perceptions. The questionnaire assesses each dimension using a single-item scale from 0 to 10.
- Perceived Stress Scale (PSS) [38]. It is a 10-item measure of the degree to which situations in one’s life are appraised as stressful. The items refer to people’s subjective appraisals of events.
- Post Traumatic Growth Inventory (PTGI) [39], a 21-item instrument for assessing positive outcomes in people who have experienced traumatic events. It measures five domains or factors: relating to others, new possibilities, personal strength, spiritual change, and a deeper appreciation of life.
- Five Facet Mindfulness Questionnaire (FFMQ) [40], a 39-item self-report questionnaire measuring five unique facets associated with the overarching construct of mindfulness disposition. The facets included are observing, describing, acting with awareness, nonjudgment, and nonreactivity.
- A clinical diary of the homework practice: it is a clinical diary regarding frequency and duration of homework assigned in the group sessions, that patients are asked to fill out every time they spent time on homework practice.

Caregivers will be asked to complete the same self-report measures administered to the patients, except for the FSS and the IPQ-Brief, which are related to the disease. Instead of FAMS, caregivers will complete the WHO-Quality of Life Brief [41].

### Intervention

Treatments will be independent and blinded from the clinical psychologists conducting the clinical assessments. The experimental group will undergo a 8 weekly sessions of 3 h each with a group based Mindfulness Based Intervention. The control group will follow the same structure as the Mindfulness Based Intervention.

#### Mindfulness based intervention (MBI)

The MBI is based on Mindfulness Based Stress Reduction (MBSR) [42] and Mindfulness Based Cognitive Therapy (MBCT [43] protocols. The MBI comprises an 8-week group program. Participants will took part in a 3 h single weekly sessions, and there will be also an additional all-day session of 7 h. Each session will cover specific exercises and topics within the context of mindfulness practice and training (body scan, breath meditation, walking meditation, yoga exercises). Participants will be required to carry out daily 45-min homework assignments, which consist on mindfulness exercises and mindfulness applications in everyday life. We will attempt to objectively verify the execution of homework practice through the clinical diary.

In order to maximize the clinical utility in people with MS suffering from depressive symptoms, we will tailor mindfulness intervention on the needs of this specific population [44].

We will apply a modified MBSR protocol integrated with techniques from Sensorimotor Psychotherapy (SP) [45], in which:

- particular emphasis will be paid to somatic resources (grounding, centering, boundaries, lengthening the spine, body self-soothing techniques, etc.) to stabilize the emotional state to prepare and sustain the mindful disposition;
- explanation of stress response will be integrated with the concept of “window of tolerance” [46], that is the range of optimal arousal which is necessary to stay in touch with the present moment experience. This concept allows a more precise awareness of the arousal state, recognizing hyper-arousal and hypo-arousal symptoms, and consequently to manage them more effectively;
- considering the attention deficits, which may be due both to neuropsychological deficits and to the depression itself, great attention is paid to attention training;
- considering interpersonal deficits in depression, interpersonal mindful listening is specifically addressed, developing the capacity to listen in a mindful state, creating a space inside to observe in a compassionate and non-judgmental way sensations, emotions and thoughts evoked by the other’s narration;
- great treatment emphasis will be paid on acceptance and self-compassion, and specific tools are used to increase it, such assessment and decentering of negative cognitions, and mindful body self- touch, becoming aware of sensations, feelings, thoughts and needs in the intimate relationship with the self.

MBI groups will be delivered by a psychotherapist with mindfulness training experience, who is experienced in working with people with MS.

#### Active control group

Our control group is based on the active control group of the study of Grossman et al. [47].

This intervention was designed to control for the non-specific elements of the MBI treatment. It will be based on a psycho-educational framework and will include some relaxation techniques.

In each session is discussed a MS-related topic. Relaxation and gentle stretching exercises will be proposed at the end of each session. Homework and the material (photocopies, slides, etc.) discussed in group sessions is left to each participant to encourage participants to practice exercises between sessions. This intervention will follow the same structure and weekly format of the MBI intervention. The only difference will be the absence of an all-day session. Each component has its counterpart in the MBI curriculum, but emphasis is placed upon not describing or training mindfulness skills to the control group. The groups will be delivered by a psychotherapist with relaxation training experience, who is experienced in working with people with MS.

#### Outcome measures and statistical analysis

Primary outcome measures will be: 1) the improvements of BDI-II score from T0 to T2; 2) the proportion of participants at T2 that do not have a BDI-II score greater than 13.

Secondary outcome measures will be the comparison of the scores of FAMS, BAI, FSS, IIP, IPQ-Brief, PSS, PTGI and FFMQ of the two groups at T0, T1 and T2. Another secondary outcome aim will be the reduction of psychological symptoms (BDI-II, BAI, IIP, PSS, PTGI) and a reduction in QoL (WHOQoL-Brief) in caregivers of patients with MS.

The efficacy of MBI will be evaluated by comparing the results to different instruments administered to patients before participating at MBI (T0), after MBI (T1) and at the 6-month follow-up (T2) and by comparing these results with those of patients who were included in the active control group. Intention to treat analyses will be used.

A longitudinal (within subjects) and cross sectional (between subjects) evaluation of our outcome measures will be performed through a repeated measures multivariate analysis of covariance (ANCOVA) using the SPSS GLM procedure. The pre-treatment value will be the covariate, and the treatment condition will be the inter-subjects factor. The intra-subject factor will be a time condition, relating to changes from post-treatment to 6-month follow-up.

The proportion of participants at T1 and T2 no longer meeting the BDI-II cut-off criteria for depression will be evaluated through the Chi square test.

#### Power calculation

The sample size calculation is based on the primary outcomes for patients with MS.

Statistical power calculation was based on previous studies [28, 48]: assuming power =0.80 and 0.05 significance, a minimal sample size of 44 patients/group will be required, based on an effect size of 0.54.

To obtain the 88 patients needed for the study, it is expected to do the screening of at least 600 patients. On the basis of the information that is available on the prevalence of depression in MS patients, on the average 25 % in previous studies, this number should guarantee there will be at least 150 participants. The number of refusals and treatment interruptions is estimated at not above 40 % which leads to about 90 cases.

## Discussion

As far as we know this trial will be the first randomized controlled trial testing the efficacy of group-based MBI for patients with MS with a comparison with an active control group with a specific focus on depressive symptoms.

Secondary outcome measures are levels of QoL, anxiety, perceived stress quality of interpersonal relationship, illness perception and fatigue.

The main strengths of this trial are: 1) a comparison with an active control group; 2) a main focus on depressive symptoms; 3) psychological symptoms are not only self-reported but our assessment includes also a clinical interview; 4) involvement and assessment of caregivers’ psychosocial functioning.

The comparison with an active control group, that is specifically designed to control for the non-specific elements of the MBI treatment and matched for duration and format of group, will permit to assess with an adequate methodology the role of mindfulness training on changes in several psychosocial and physical measures.

The focus on depressive symptoms is supported from what is stated by the Goldman Consensus Conference [12] and by the American Academy of Neurology [13] that highlight how depression is under-diagnosed and under-treated in people with MS and can lead to a decrease in adherence to medication as well as a reduced QoL. MBIs have been proven effective in improving depressive symptoms and in enhancing QoL in many different chronic conditions and recently also in patients with MS. MBIs can therefore be useful in counteracting the suffering caused by the symptoms, the fatigue, the depressive and anxiety symptomatology and the distress in dealing with the disability.

Previous studies did not use objective measures of psychosocial functioning of patients before and after the intervention, and their findings were based only upon outcome measures reported by patients. In this study we propose to assess patients’ psychological functioning with a semi-structured clinical interview (SCID) in addition to the self-reported measures.

Furthermore, our project aims to involve patients’ caregivers. We expect that caregivers will benefit from the treatment received, taking into consideration the load of stress and difficulties related to the management of a chronic disabling diseases like MS. In fact, a recent study has shown that patients’ depressive state influences the deterioration of QoL and the depression of caregivers [49].

Finally, our project is based on MBSR and MBCT protocols, but in order to maximize the clinical utility in people suffering from MS and depression we tailored mindfulness intervention on the needs of this specific population [44]. We will apply a modified MBSR protocol integrated with body centered techniques from Sensorimotor Psychotherapy [45]. SP was developed by Pat Ogden on the model of Ron Kurtz, a body psychotherapy built on mindfulness [50]. Most of these points are already part of the MBSR and MBCT model, but in this integration they are deepened more specifically to increase clinical efficacy.

Results of this trial will provide methodologically grounded data to consider the applicability of MBIs in the usual practice of MS care centers and can help to broad treatment possibilities for patients with MS-related psychological distress.

This study has some potential limitations. The main limitation of the project is the exclusion of patients with a severe disability due to the characteristics of the intervention, which requires a mobility at least partially preserved. In addition, no neuroimaging and neurobiological assessments are planned in the project.

Still and in conclusion, we deem that this study may be the first clinical controlled trial in which MBI will be investigated in comparison with an active control group in patients with MS.

## Abbreviations

MS: Multiple Sclerosis
QoL: Quality of Life
CBT: Cognitive Behavioural Therapy
MBI: Mindfulness Based Intervention
BDI-II: Beck Depression Inventory
EDSS: Expandend Disability Status Scale
SCID: Structured Clinical Interview-DSM-IV
FAMS: Functional Assessment of Multiple Sclerosis
BAI: Beck Anxiety Inventory
FSS: Fatigue Severity Scale
IIP: Inventory of Interpersonal Problems
IPQ-Brief: Brief Illness Perception Questionnaire
PSS: Perceived Stress Scale
PTGI: Posttraumatic Growth Inventory
FFMQ: Five Facet Mindfulness Questionnaire
ANCOVA: Analysis of covariance
MBSR: Mindfulness Based Stress Reduction
MBCT: Mindfulness Based Cognitive Therapy
SP: Sensorimotor Psychotherapy
